# Gut-Brain-Microbiota Axis: Antibiotics and Functional Gastrointestinal Disorders

**DOI:** 10.3390/nu13020389

**Published:** 2021-01-27

**Authors:** Tarkan Karakan, Ceren Ozkul, Esra Küpeli Akkol, Saniye Bilici, Eduardo Sobarzo-Sánchez, Raffaele Capasso

**Affiliations:** 1 Department of Gastroenterology, Faculty of Medicine, Gazi University, 06570 Ankara, Turkey; 2 Department of Pharmaceutical Microbiology, Faculty of Pharmacy, HacettepeUniversity, Sıhhiye, 06110 Ankara, Turkey; cerenozkul@hacettepe.edu.tr; 3 Department of Pharmacognosy, Faculty of Pharmacy, Gazi University, Etiler, 06330 Ankara, Turkey; esrak@gazi.edu.tr; 4 Department of Nutrition and Dietetics, Faculty of Health Science, Gazi University, Beşevler, 06560 Ankara, Turkey; sgbilici@gazi.edu.tr; 5 Instituto de Investigación y Postgrado, Facultad de Ciencias de la Salud, Universidad Central de Chile, 8330507 Santiago, Chile; eduardo.sobarzo@ucentral.cl; 6 Department of Organic Chemistry, Faculty of Pharmacy, University of Santiago de Compostela, 15782 Santiago de Compostela, Spain; 7 Department of Agricultural Sciences, University of Naples Federico II, 80055 Portici, Naples, Italy

**Keywords:** gut microbiota, gut microbiome, gut-brain axis, functional bowel disorders, irritable bowel syndrome, antibiotics, probiotics

## Abstract

Gut microbiota composition and function are major areas of research for functional gastrointestinal disorders. There is a connection between gastrointestinal tract and central nervous system and this is mediated by neurotransmitters, inflammatory cytokines, the vagus nerve and the hypothalamic-pituitary-adrenal axis. Functional gastrointestinal disorders are prevalent diseases affecting more than one third of the population. The etiology of these disorders is not clarified. Visceral hyperalgesia is the main hypothesis for explaining clinical symptoms, however gut-brain axis disorder is a new terminology for functional disorders. In this review, microbiota-gut-brain axis connection pathways and related disorders are discussed. Antibiotics are widely used in developed countries and recent evidence indicates antibiotic-induced dysbiosis as an important factor for functional disorders. Antibiotics exert negative effects on gut microbiota composition and functions. Antibiotic-induced dysbiosis is a major factor for occurrence of post-infectious irritable bowel syndrome. Cognitive and mood disorders are also frequent in functional gastrointestinal disorders. Animal and human trials show strong evidence for the causal relationship between gut microbiota and brain functions. Therapeutic implications of these newly defined pathogenic pathways are also discussed.

## 1. Introduction

The human microbiota is a collection of microorganisms that live in and on us. Over the past two decades, we have had an enormous amount of publications about gut microbiota in health and disease. Improvement in sequencing technologies coupled with bioinformatics science are making this research more feasible and cheaper. For instance, the estimated ratio of human:microbiota cells was recently revised from 10 times to 1.3 times more than human cells [1,2]. Even this 1.3 ratio (microbiota cells/human cells) is mind-blowing since these cells harbor their own genetic material. When we make a comparison between the number of genes between human and microbes in our body, over 99% of the genes in our body are microbial (numbering over 10 million). In the evolution process, we co-evolved with microbiota and they might have influenced our immune system and epigenetics [3,4,5,6,7]. Although we can modulate the expression of human genome through diet and lifestyle, human microbiome can also be directly modulated by these factors. Therefore, from a therapeutic perspective, gut microbiota can be manipulated by diet, drugs, lifestyle, etc. This opportunity gives us a new horizon for therapeutic aspect of chronic diseases. In the last decade, we had great progress in the field of molecular genetic tests and today there are evolving methods of analyzing gut microbiome [8].

Functional gastrointestinal disorders (FGID) are prevalent diseases affecting more than one third of the population [9]. Emerging evidence suggests a new definition for these disorders, involving gut-brain axis [10]. These syndromes are also pathogenetically related to disorders of gut-brain interaction [11]. Rome Criteria defines 26 distinct adult FGIDs, most well-known are irritable bowel syndrome (IBS) and functional dyspepsia (FD). The pathogenesis of these disorders is not well-defined [12] and these disorders are associated with psychiatric co-morbidities, including panic attack, anxiety disorder, depression, hypochondriac behavior and somatization [13,14,15,16,17,18]. Recent studies showed that these behavioral changes are related to disturbances in gut-brain axis, including gut microbiota [19].

### Methodology

A search for peer-reviewed articles published before September 2020 was performed in PubMed, Google, Web of Science, and MEDLINE to identify studies observing the effects of antibiotics in gut-brain-microbiota axis. Search terms used were (“gut” or “gut-brain” or “antibiotic”) or and (“microbiota”) and (“gastrointestinal disorder”). All titles/abstracts of the occasioning articles were appraised manually. For relevant abstracts, full articles were achieved and reviewed.

## 2. Gut-Brain-Microbiota Axis

**Figure 1:**
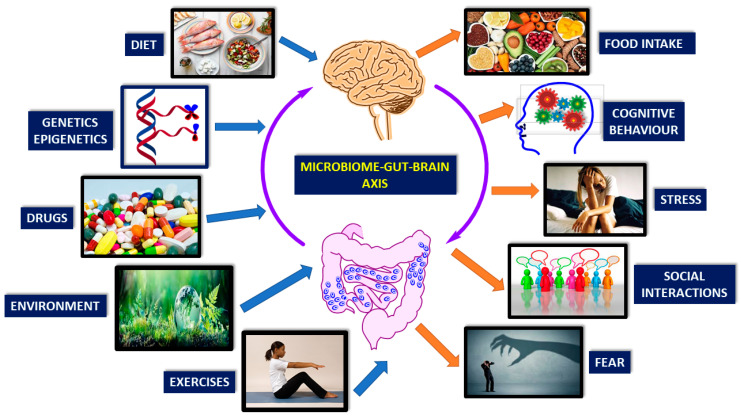
Brain-gut-microbiome axis is a dynamic, interactive network. Factors affecting the communication of these elements are mainly genetics, diet, and lifestyle (Modified from reference [8]).

The brain-gut-microbiota axis is a bidirectional system enabling gut microorganisms to communicate with the central nervous system (CNS), and the CNS with the gut [19]. The mechanisms of signal transmission are complex and not fully understood, but include neural, endocrine, immune and metabolic pathways [19]. There are many factors affecting microbiome-gut-brain-axis. These are diet, genetics, drugs, environment, exercise, cognitive behavior, stress, social interactions, and fear (Figure 1) [20,21,22,23,24,25,26,27,28].

Gut microbes are capable of producing most neurotransmitters found in the human brain. While these neurotransmitters primarily act locally in the gut, modulating the enteric nervous system, there is also undeniable evidence indicating that gut microbes can influence CNS through multiple mechanisms. The treatment with probiotic *Bifidobacteria* for instance, can increase the amount of tryptophan, the precursor of serotonin [29]. Some *Lactobacilli* species alter gamma-aminobutyric acid (GABA) metabolism and change brain GABA receptor expression and behavior [30]. Preclinical studies show that the vagus nerve is the main route for exerting the effects of gut microbiota on CNS. *Lactobacillus rhamnosus* has a central effect in animals and this was ameliorated by vagotomy [31]. In fact, patients with a history of vagotomy have diminished risk for certain neurological diseases [32]. Synthesis and release of neurotransmitters from bacteria have been reported: *Lactobacillus* and *Bifidobacterium* species can produce GABA; *Escherichia, Bacillus* and *Saccharomyces* spp. can produce noradrenaline; *Candida, Streptococcus, Escherichia* and *Enterococcus* spp. can produce serotonin; Bacillus can produce dopamine; Lactobacillus can produce acetylcholine [33,34,35]. Although these neurotransmitters can cross inflamed intestinal mucosal barrier, they cannot cross blood–brain-barrier (BBB) in healthy conditions. Another way of gut-brain interaction is the stimulation of hypothalamic-pituitary-adrenal (HPA) axis, which induces cortisol secretion. This system is the main stressor system in the body and it is mainly regulated by gut-HPA axis [36,37,38,39,40,41,42,43,44,45,46,47]. Psychological or physical stress can affect HPA axis and subsequently gut microbiota/barrier function (e.g., IBS) [33].

Post-infectious IBS is a prototype for gut-brain axis disorders. Water-born gastroenteritis outbreak occurred in United States and people affected from this *E.coli* infection later developed IBS-like symptoms including co-morbid depression, anxiety disorder [48].

## 3. Pathways of Communication

**Figure 2:**
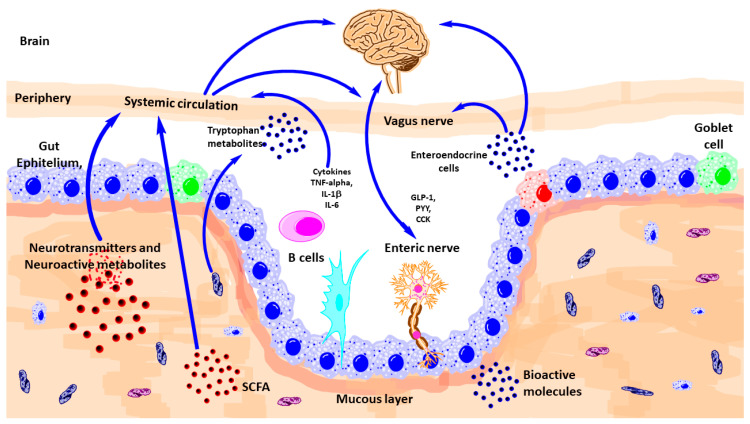
Schematic outlining the various known pathways of communication between the gut-microbiota and the brain. CCK, Cholecystokinin; GLp-1, glucagon-like peptide-1; IL, interleukin; PYY, peptide YY; TNF, tumor necrosis factor; SCFA, short-chain fatty acid. (Modified from reference [8]).

There are diverse ways of communication between gut microbiota and brain such as autonomic nervous system, vagus nerve, enteric nervous system, neurotransmitters, and immune system (Figure 2).

### 3.1. Autonomic Nervous System (ANS)

The autonomic nervous system (ANS) comprises the sympathetic and parasympathetic branches. Combined with activity from the enteric nervous system (ENS) and influenced by the CNS, the ANS is responsible for physiological homeostasis, as well as responding to endocrine, motor, autonomic, and behavioral areas. Gut microbiota communicate with ANS bidirectional both antagonistic and synergistically [49,50]. Afferent nerves carry information from visceral organs to CNS and from CNS, important survival messages sent towards peripheral organs. ANS acts as the most immediate responder in health and disease states [51,52,53,54,55,56,57,58]. Local GI autonomic activation can be stimulated by afferent feedback loops from the microbiota and CNS efferent modulation [59]. Microbiota-related metabolites such as tryptophan (and end products, e.g., serotonin-5HT), GABA, catecholamine’s mediate ANS related effects. Sympathetic innervation has post-ganglionic vasoconstrictor effects and also suppressive effects on gut secretions and motility. Intestinal mucus layer is regulated by sympathetic innervation, by modulating mucosal immune system, microbial composition, and function [52,54,60].

### 3.2. Vagus Nerve

It is the tenth cranial nerve and the longest in the body with extensive connections and networks with peripheral organs. The vagus exerts anti-inflammatory actions via medullary dorsal motor nucleus. The vagal modulation of macrophage action an important factor for the inflammation in inflammatory bowel disease (IBD) [53].

### 3.3. Enteric Nervous System (ENS)

At the interface of microbiota and host, there is a network of gut neurons called ENS. Anatomically divided as submucosal and myenteric plexus, ENS regulates gut motility and secretions [61]. Factors affecting neurodevelopment and health status of CNS may also affect ENS integrity. Gut microbiota influences development and function of ENS via pattern recognition receptors (PRR) and including Toll-like receptors (TLRs), especially TLR-2 and TLR-4. These TLRs are involved in the recognition of microbial molecules [62]. *Bacteroides fragilis* and the capsular exopolysaccharide, are good examples, which can influence ENS function [63]. *L. rhamnosus* strain (JB-1) performs this action via a G protein-coupled receptor-mediated pathway [31]. Recent data showed that stress-induced alterations in ENS activity, via stimulated acetylcholine release, were influenced by both maternal separation and the microbiota [64]. This proves that the microbiota may affect ENS-related gut dysfunction associated with early-life stress. ENS abnormalities are associated with life-threatening GI disorders including Hirsch sprung disease and chronic intestinal pseudo-obstruction [65]. Moreover, the ENS is also involved in disorders of the CNS, including ASD, Alzheimer’s disease (AD), and Parkinson’s disease (PD) [66].

### 3.4. Immune System

Gastrointestinal tract has the highest number of immune cells in the body and there is a delicate, complex communication with the gut microbiota [67]. Mucus produced by epithelial Goblet cells provide a barrier against contact with host cells and microbial elements. The gut microbiota influences regulation of subsets of immune cells including T helper (Th), T regulatory (Treg), natural killer (NK), mononuclear phagocytes, and innate lymphoid cells [68]. The mechanisms by which the gut microbiota influences innate and adaptive responses during health and disease is still being investigated.

**Figure 3:**
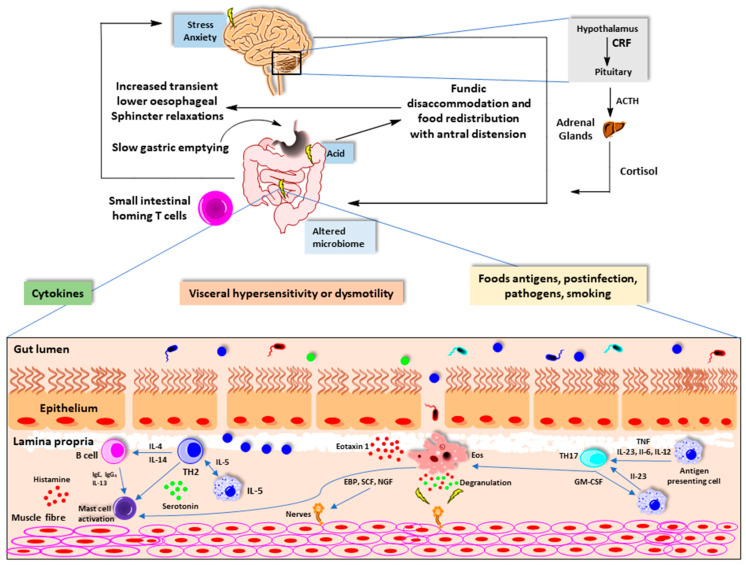
Mucosal immune system activation and functional gastrointestinal disorders. (Adapted from reference 19). CRF, corticotrophin-releasing factor; ACTH, adrenocorticotropic hormone; Ig, immunoglobulin; IL, interleukin; TH, T-helper cell.

Environmental factors affect immune function. The release of cytokines promotes the activation and recruitment of eosinophils, B cells, and mast cells. In addition to the traditional T-helper 2 pathway, secretion of IL-23 from antigen-presenting cells, such as dendritic cells, B cells, and macrophages, promotes T helper-cell 17 differentiation. Degranulation of these cells disrupt intestinal barrier and enteric nerves. This results in visceral hypersensitivity and dysmotility [19]. α4β7 gut homing T cells are a marker of intestinal inflammation in both functional dyspepsia and irritable bowel syndrome. The site and extend of intestinal immune activation can define the phenotype such as functional heartburn, functional dyspepsia, irritable bowel syndrome, functional constipation, or functional diarrhea [19] (Figure 3).

## 4. Impact of Antibiotic Use on Gut Microbiome-Brain Axis

The bi-directional communication between gut microbiota and brain ensures the maintenance of intestinal homeostasis and likely affects CNS functions in different time points of life, from early life neurodevelopment to aging and neurodegeneration [66]. Studies on the effect of antibiotics against gut microbiota-brain axis should be interpreted by considering the well-defined age-related differences in gut microbiota composition and diversity as well as its resilience and stability [69]. Moreover, the effect of antibiotics may be specific to an antibiotic group, course, and duration. Their absorption from GI tract is also critical in order to evaluate the direct effect of gut microbial perturbations without the neurologic effect of antibiotic itself [70]. For instance, metronidazole is a widely prescribed absorbable antibiotic for GI disorders, which can cross the blood–brain barrier [71]. Non-absorbable antibiotics such as vancomycin does not cross blood–brain barrier and become concentrated in the gastrointestinal tract, excluding the direct effect of antibiotics on CNS [72]. Route of antibiotic administration is also another important factor in experimental gut-brain axis studies, since oral gavage may induce stress in animals and may lead to misinterpretation of behavioral parameters [73].

Initial colonization of the gut begins in early life and mainly shaped by gestational age [74], mode of delivery (vaginal birth vs. Caesarean section) [75], method of feeding (breastfeeding vs. formula feeding) [76], and antibiotic exposure [77]. The early postnatal period is critical for microbial community and immune cell development. During this critical window, morphological and functional development in CNS also takes place, which is likely to be directly or indirectly influenced by gut microbiota [78,79]. A growing body of evidence from both epidemiological [80,81,82] and experimental studies [71,83,84] indicates that the gut microbiota has a crucial role in modulating brain function and behavior via gut-brain axis.

The majority of the studies providing evidence for the role of the gut microbiota in neurodevelopment come from germ-free animal experiments including altered hippocampal neurogenesis and decreased expression of brain-derived neurotrophic factors in germ-free mice [66,85,86]. Unlike germ-free conditions, antibiotics can selectively deplete microbial populations which is advantageous to design flexible experimental models and to determine targeted approaches for specific bacterial taxa [73]. Disruptive effect of antibiotic use especially in early life has known to cause long lasting consequences on microbial community and immunity, which may lead to increased predisposition and susceptibility to further disease progression such as IBD [77,87]. Majority of the studies using broad spectrum antibiotic cocktails showed comparable results with those observed in germ-free mice involving impaired social behaviors, neurogenesis and cognitive function together with the perturbations in gut microbiota [88,89].

In addition to evidence from experimental studies, a recent longitudinal population-based study reported that early-life antibiotic exposure is associated with an increased risk for psychiatric disorders [81]. Another recent clinical study also showed an altered auditory processing and recognition memory responses in infants who received intravenous antibiotics after delivery, supporting the importance of microbiota-gut-brain axis in humans during early life [80]. As a neurodevelopmental condition, autism spectrum disorder has also been linked to gut microbiota-brain axis dysfunction [90]. According to a recent meta-analysis, prenatal antibiotic exposure significantly increased the ASD risk in children. Interestingly, non-absorbable antibiotic vancomycin treatment, which mainly target Gram positive bacteria such as *Clostridium* spp., resulted in improved eye contact behavior and less constipation in autistic children [82].

Although not specifically focused in this review, neurodegenerative disorders such as Alzheimer’s disease (AD) are another consequence of gut microbiota-brain axis impairment. Minter et al. [78] reported that broad-spectrum postnatal antibiotic use reduced the amyloid plaque deposition in aged APPSWE/PS1ΔE9 transgenic mice suggesting that selective, targeted microbiome-based treatments can be used in early stage AD. A possible potential mechanism might be blooming in Lachnospiraceae family, one of the main butyrate-producers of the gut microbiota, after antibiotic treatment [91]. The increased butyrate production may induce T-reg differentiation, which has beneficial effect in AD pathogenesis by modulating microglial response amyloid-β deposition [92].

In addition to early life being of critical importance in terms of disruptive antibiotic effect on gut microbiota, antibiotic studies on adult animals also showed impaired behavior and brain function [71,83,93,94,95] and neurodegenerative disorders [96] both in prolonged and short courses, supporting the crucial role of gut microbiota-brain axis during lifespan. Some of the studies showed that these disruptive effects can be rescued by probiotic administrations [89,97].

Experimental reports summarizing the effect of antibiotics by considering the type of antibiotic, duration, route and age of exposure on gut-brain axis are depicted in Table 1. These experimental studies can be evaluated by considering broad-spectrum antibiotic cocktail strategies, which can broadly target gut microbiota including anaerobic bacteria causing germ-free like conditions [73], and targeted therapies using specific antibiotic groups such as Vancomycin. Both approaches lead to brain and behavioral impairments suggesting the role of disruption in both structure and function of the microbiota together, rather than a specific taxonomic group. A study by Erny et al. [98] also highlights the importance of gut microbial metabolites in microglia maturation and function. They showed that gut microbiota depletion by antibiotics resulted in defective microglia, which can be restored by microbiota-derived metabolites such as SCFA. Accordingly, antibiotic-driven disruption in gut microbiota may cause perturbation in functional gut microbiota, leading to alterations in microbial metabolites such as SCFA, as possible regulators of CNS inflammation [99].

While majority of the experimental studies in Table 1 highlight the complex role of gut microbiota in brain function both in early-life and adult age, the study by Minter et al. suggested a potential protective role of postnatal antibiotics in further AD progression, which provides a mechanism for Aβ deposition as a consequence of gut microbiota driven neuroinflammation [78].

## 5. Conclusions

Gut microbiota plays a vital role in the pathogenesis of functional gastrointestinal disorder. Gut microbiota and brain interactions are important factors for prevention and therapy. Further clinical studies are required for understanding the true effect of gut microbiota on disease progression in humans. While antibiotics are essential treatment strategies in most conditions, the long-lasting effects on host microbiome and immune functions, especially during early life should be interpreted with caution. We need prospective randomized trials for the therapeutic potential of gut microbiota modulation in functional gastrointestinal disorders.

## Figures and Tables

**Table 1:** Summary of studies demonstrating the effect of antibiotics (abx) on gut-brain axis in early life and adulthood.

Condition	Model	Type of abx	Duration	Age and Route of abxExposure	Effects	Conclusion	Ref.
Behavior and brain function	NIH Swiss mice	Broad spectrum abx cocktail (ampicillin, vancomycin, neomycin, metranizdazole, amphotericin-B)	60 days	Postnatal, post-weaning day 21–80 (continuous treatment) via drinking water	Alterations in gut microbiota composition Abx reduced anxiety, induced cognitive deficits, altered dynamics of the tryptophan metabolic pathway Abx significantly reduced BDNF, oxytocin and vasopressin expression in the adult brain	Dysregulation of the gut-brain axis in the post-weaning period may contribute to the pathogenesis of disorders associated with altered anxiety and cognition.	[88]
Behavior and brain function	C57BL/6 mice	*Per os* Ampicillin, Meropenem, Neomycin, Vancomycin	11 days	8–11 weeks old mice exposed via oral gavage	Alterations in gut microbiota composition Impaired object recognition but not spatial memory. Gut microbiota disruption alters the metabolic profiles in colon and plasma. The cerebral neuropeptide Y system is severely disturbed in gut dysbiosis	Circulating metabolites and the cerebral neuropeptide Y system play an important role in the cognitive impairment and dysregulation of cerebral signaling molecules due to abx-induced gut dysbiosis.	[71]
Behavior and brain function	BALB/c mice	Nonabsorbableabx Nneomycin, Bacitracin, Primacin	7 days	6–8 weeks old mice exposed via drinking water	Alterations in gut microbiota composition Increased hippocampal BDNF and exploratory behavior	Gut microbiota influences brain chemistry and behavior independent from the autonomic nervous system, gastrointestinal-specific neurotransmitters, or inflammation	[83]
Behavior and brain function	C57BL/6 mice	Drinking water Broad spectrum abx cocktail (ampicillin-sulbactam, vancomycin, ciprofloxacin, imipenem-cilastatin, metronidazol	7 weeks	6–8 weeks old mice exposed via drinking water	Decreased hippocampal neurogenesis and memory retention Observed effects were mediated by Ly6Chi monocytes	Abx decrease neurogenesis and cognitive function	[89]
AD	APP_SWE_/PS1_ΔE9_ mice	Abx cocktail (gentamicin, vancomycin, metronidazole, neomycin, ampicillin kanamycin, colistin and cefaperazone)	7 days	Postnatal days 14–21 (pre-weaning) via oral gavage	Alterations in gut microbial diversity Upregulated circulating Treg cells Reduced Aβ plaque deposition Increased soluble Aβ Reduced Plaque-localized gliosis	Protective effect of post-natal abx on further AD progression	[78]
Behavior and brain function	C57BL/6 Male mice	Ampicillin Streptomycin Clindamycin	2 weeks	6 weeks old mice exposed via drinking water	Alterations in gut microbiota composition Behavioral changes, including increased immobility in the tail suspension test and reduced social recognition Altered BDNF/TrkB signaling Increased number of activated microglial cells in hippocampus Lachnospiraceae and endocannabinoids levels correlate negatively with behavioral changes.	Abx-perturbed microbiota leads to a depressive-like behavior and impaired social activity associated with biochemical and functional changes in the hippocampus	[93]
Behavior and brain function	Sprague Dawley rats	Abx cocktail (ampicillin vancomycin, ciprofloxacin, imipenem and metrondiazole)	13 weeks	9 weeks old rats exposed via drinking water	Altered miRNA expression profile in the amygdala and prefrontal cortex of abx-treated mice	Gut microbiome is crucial for appropriate regulation of miRNA expression in brain regions implicated in anxiety-like behaviors	[94]
Behavior and brain function	BALB/c mice	Ceftriaxone	11 weeks	6–8 weeks old mice exposed via oral gavage	Decreased gut microbiota richness and diversity Increased anxiety-like, depression-like, and aggressive behaviors	Abx-perturbed microbiota could affect the nervous system, influencing brain function.	[95]
Parkinson’s Disease (PD)	Thy1-α-synuclein mice	Drinking water Ampicillin Vancomycin Neomycin Gentamycin Erythromycin	7 days	5 weeks old mice exposed via drinking water	Abx treatment ameliorates, while microbial re-colonization promotes, PD pathophysiology in adult animals Abx-treated mice display decreased αSyn-dependent motor dysfunction	Gut microbiota regulate movement disorders in mice and gut microbiota alterations represent a risk factor for PD	[96]
Behavior and brain function	BALB/c mice	Penicillin V (low-dose)	7 days	Postnatal days 14–21 (pre-weaning) via oral gavage	Long-term sex-dependent changes in social behavior, but not anxiety-like traits, in male mice only Changes were associated with decreased hippocampal expression of AVPR1A and AVPR1B *L. rhamnosus* supplementation together with abx treatment can prevent behavior changes	Post-natal exposure to a clinically relevant dose of abx has long-term, sex dependent effects on the CNS and may have implications for the development of neuropsychiatric disorders	[97]
Behavior and brain function	Wistar rats	Non-absorbable abx SST	60 days	Prenatal abx was administered starting before breeding to gestational age 15 via food	Offspring showed reduced social interactions at postnatal day 25 and increased anxiety at postnatal day 35	Maternal exposure to SST leads to alterations in offspring behavior	[100]
Behavior and brain function	BALB/c mice	Penicillin V (low-dose)	28 days	Prenatal abx was administered to pregnant mother via drinking water	Alterations in gut microbiota composition No effect on locomotor activity at postnatal week 6 Increased aggression and decreased social avoidance behavior Increased Avpr1b and cytokine expression in the frontal cortex Increased tight junction protein levels in the hippocampus Effects could be partially prevented by *Lactobacillus rhamnosus* JB-1	Early-life low dose abx exposure induced long-lasting changes in gut microbiota, neuroinflammation and behavior	[84]
Behavior and brain function	C57BL/6 mice	Nonabsorbableabx: Neomycin Basitracin Pmaracin	7 days	Prenatal abx was administered to pregnant mothers via drinking water	Decreased Lactobacillales and increased *Clostridium*XIVa in offspring born from abx-treated dams Decreased locomotor activity in offspring born from abx-treated dams The behavioral phenotypes of the offspring from abx-treated dams at postnatal week 4 could be partially rescued through cross-fostering	Administration of non-absorbable abx to pregnant dams to perturb the maternal gut microbiota during pregnancy leads to alterations in the behavior of their offspring	[101]
Behavior and brain function	Sprague Dawley rat	2 abx strategies: -Vancomycin -Nonabsorbableabx Pimaricin, Bacitracin, Neomycin	9 days	Postnatal days4–13 via drinking water	Postnatal vancomycin significantly altered the microbiota Increased visceral hypersensitivity in adulthood of male rats for both vancomycin and abx cocktail treated rats Both abx treatments had no effect on anxiety related behavior	Early-life temporary disruption of the gut microbiota results in very specific and long-lasting changes in visceral sensitivity in male rats, a hallmark of stress-related functional disorders of the brain–gut axis such as irritable bowel disorder.	[102]
Gut neuromuscular function	C57BL/6 mice	Broad spectrum abx cocktail (vancomycin, neomycin, ampicillin, metranizdazole)	14 days	3 weeks old mice exposed via oral gavage	Abx lead to impaired GIS motility, impaired bowel architecture, altered excitatory neuromuscular contractility	Intestinal microbiota is crucial for enteric nervous system to maintain proper gut neuromuscular function	[103]

abx: antibiotic, AD: Alzheimer’s disease, Avpr1: arginine vasopressin receptor 1B, BDNF: brain-derived neurotrophic factor, CNS: Central nervous system, GIS: gastrointestinal system, PD: Parkinson’s disease, SST: SuccinylSulfaThiazole.
